# Exploring the Role of Cell Wall-Related Genes and Polysaccharides during Plant Development

**DOI:** 10.3390/plants7020042

**Published:** 2018-05-31

**Authors:** Matthew R. Tucker, Haoyu Lou, Matthew K. Aubert, Laura G. Wilkinson, Alan Little, Kelly Houston, Sara C. Pinto, Neil J. Shirley

**Affiliations:** 1 School of Agriculture, Food and Wine, Waite Research Institute, The University of Adelaide, Glen Osmond, SA 5062, Australia; haoyu.lou@adelaide.edu.au (H.L.); matthew.aubert@adelaide.edu.au (M.K.A.); laura.g.wilkinson@adelaide.edu.au (L.G.W.); alan.little@adelaide.edu.au (A.L.); neil.shirley@adelaide.edu.au (N.J.S.); 2 Australian Research Council Centre of Excellence in Plant Cell Walls, The University of Adelaide, Glen Osmond, SA 5062, Australia; 3 Cell and Molecular Sciences, The James Hutton Institute, Dundee DD2 5DA, UK; Kelly.Houston@hutton.ac.uk; 4 Departamento de Biologia, Faculdade de Ciências da Universidade do Porto, 4169-007 Porto, Portugal; sarapintomendes94@gmail.com

**Keywords:** cell wall, polysaccharide, development, glycosyltransferase, glycosyl hydrolase, differentiation, shoot meristem, root meristem

## Abstract

The majority of organs in plants are not established until after germination, when pluripotent stem cells in the growing apices give rise to daughter cells that proliferate and subsequently differentiate into new tissues and organ primordia. This remarkable capacity is not only restricted to the meristem, since maturing cells in many organs can also rapidly alter their identity depending on the cues they receive. One general feature of plant cell differentiation is a change in cell wall composition at the cell surface. Historically, this has been viewed as a downstream response to primary cues controlling differentiation, but a closer inspection of the wall suggests that it may play a much more active role. Specific polymers within the wall can act as substrates for modifications that impact receptor binding, signal mobility, and cell flexibility. Therefore, far from being a static barrier, the cell wall and its constituent polysaccharides can dictate signal transmission and perception, and directly contribute to a cell’s capacity to differentiate. In this review, we re-visit the role of plant cell wall-related genes and polysaccharides during various stages of development, with a particular focus on how changes in cell wall machinery accompany the exit of cells from the stem cell niche.

## 1. Introduction

As plant cells divide away from apical meristems, their molecular and biochemical profiles change. At the molecular level, cells adopt identities through changes in their nuclear morphology, genomic landscape, and transcriptional signatures. Changes also occur at the periphery of the cell, most notably in the abundance and organization of cell wall components such as cellulose, non-cellulosic polysaccharides, phenolic acids, lipids, and proteins [1]. Sometimes this results in terminal differentiation, for example in vascular tissues such as lignified mature fibers [2]. Changes in wall composition influence the downstream function of cells as storage units, structural networks, and solute transporters [3]. In many cases, differentiation also influences the capacity of cells to respond to stresses imparted through pathogens and the environment [4].

Despite its importance for growth and reproduction, plant cell differentiation is infrequently irreversible [5]. Many plant cells, not only those located in the meristems, possess the remarkable ability to adopt new identities. This can be a simple switch in identity between adjoining cells; for example, in the developing maize seed (kernel), where aberrant inward (periclinal) divisions of aleurone cells at the periphery result in one daughter cell retaining aleurone identity and the other adopting inner starchy endosperm identity [6]. The same thing can occur in more complex systems such as apomictic (asexual) plants, where ovule cells that adjoin normal sexual cells can spontaneously adopt germline-like identity and initiate a form of gametophyte development [7,8]. However, the plant meristem remains the epitome of differentiation capacity; meristematic stem cells can give rise to many different cell types, often referred to as pluripotency (the ability to either give rise to all cells and tissues in an organ) or totipotency (the ability to give rise to the entire organism) [9]. At a fundamental level, this indicates that fate is not fixed, and plant cells must maintain flexible cellular properties compatible with differentiation.

Much of our knowledge regarding cell differentiation has come from in vitro studies involving tissue culture, during which plant cells can be induced to de-differentiate (essentially reverse differentiation and lose specialized characteristics [10]), forming protoplasts or callus [11]. Somewhat similar to pluripotent stem cells, these totipotent undifferentiated cells can be stimulated to give rise to entire new tissues and eventually whole plants, depending on the correct exogenous application of growth hormones and vitamin supplements. Importantly, one component of in vitro de-differentiation appears to be modification or removal of the cell wall from the progenitor cell [12,13]. Moreover, in some cell types, the over-accumulation of specific cell wall components even appears to prevent de-differentiation or regeneration [14,15]. Therefore, variation in cell wall composition may contribute to the maintenance of cellular identity in some cases, while promoting the capacity for differentiation in others. How this is determined has yet to be addressed in sufficient detail, since it requires a thorough qualitative and quantitative assessment of cell wall composition at the single cell level.

**Figure 1:**
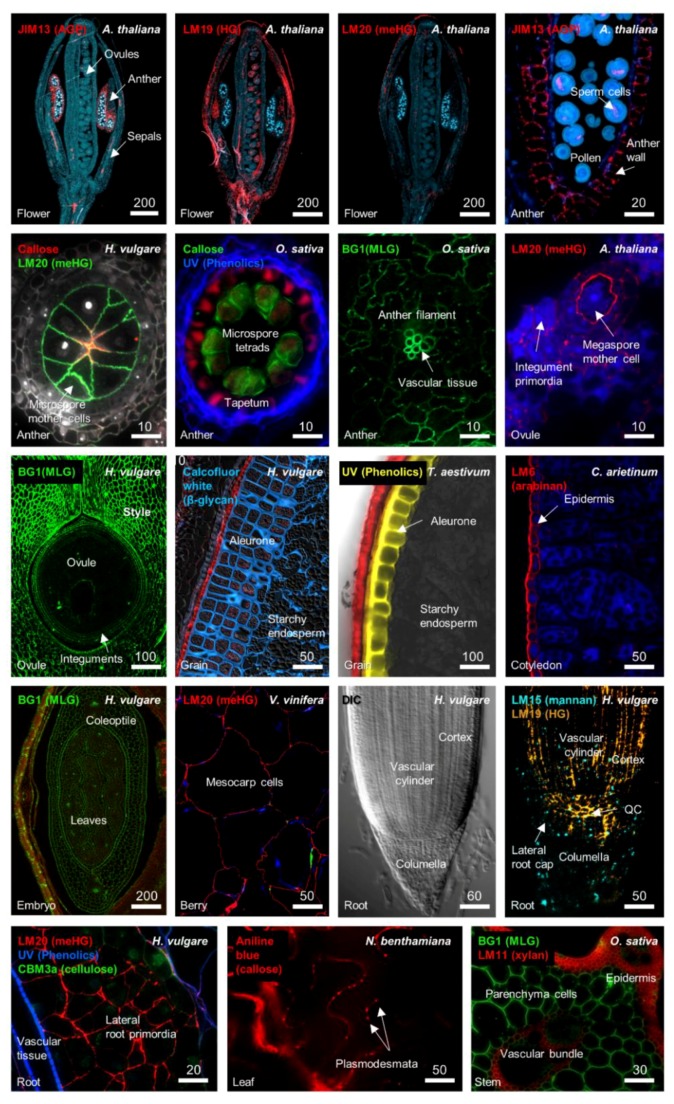
Detection of different cell wall components in distinct tissues of *Arabidopsis thaliana*, *Hordeum vulgare* (barley), *Oryza sativa* (rice), *Cicer arietinum* (chickpea), *Vitis vinifera* (grape), *Nicotiana benthamiana* (tobacco), and *Triticum aestivum* (bread wheat). The tissue origin of each section is indicated at the bottom left of each panel. The antibody or stain is indicated at the top left of each panel. Labelling of polymers was achieved through the use of diverse antibodies including BG1 (1,3;1,4-β-glucan), JIM13 (arabinogalactan proteins, AGP), LM19 (homogalacturonan, HG), LM20 (methylesterified homogalacturonan, meHG), callose (1,3-β-glucan), LM15 (mannan), LM6 (arabinan), LM11 (arabinoxylan), and CBM3a (cellulose), or stains such as aniline blue (1,3-β-glucan) and Calcofluor White (β-glycan), or UV autofluorescence. Differential contrast (DIC) microscopy was used to image the barley root tip and is shown as a reference for the adjoining immunolabelled sample. Images were generated for this review, but further details can be found in previous studies [23,29,30,31,32]. Scale bar dimensions are shown in µm.

Prevailing models suggest that there are two types of walls in plants; primary cell walls are relatively thin and flexible and are synthesized during cell growth and division, while secondary cell walls provide strength and rigidity in tissues that are no longer growing [16,17]. In general, the plant cell wall comprises a framework of cellulose microfibrils coated in diverse non-cellulosic polysaccharides. Xyloglucan (XyG) is proposed to cross-link cellulosic microfibrils, while pectins such as homogalacturonan (HG) and rhamnogalacturonan (RG) form a structurally diverse glue that provides flexibility or stiffness depending on chemical modifications [18,19]. Other classes of polymers include 1,3-β-glucan, 1,3;1,4-β-glucan, mannan, arabinan, xylan, and phenolic compounds such as lignin, which vary depending on the cell type, species, and developmental age, and appear to fulfil diverse roles [20,21,22,23]. Figure 1 shows thin sections from a number of dicot and monocot tissues labelled with cell wall-related antibodies and/or viewed under UV light, highlighting the diversity of polysaccharides present in growing tissues, as well as specific differences between organs, tissues, and individual cell types. How the different polymers interact within the cell wall matrix is constantly being revisited; direct covalent connections have been reported between pectin and xylan [24], xylan and lignin [25], and xyloglucan and cellulose [26]. However, the nature of the cross-linkages and hydrophobic interactions within the wall are not fully understood, and present significant challenges for the prediction and modelling of cell wall physicochemical properties [27]. Additional complexity is conveyed through glycoproteins such as arabinogalactan proteins (Figure 1), and other cell wall proteins such as proline-rich proteins, extensins, and expansins [28].

Classical studies in two-celled embryos of the alga *Fucus* [33] showed that there is a direct role of the cell wall in maintaining cellular fate. Extending this hypothesis to examine the role of the cell wall during differentiation of specialized cells and tissues of higher plants has proved challenging, partially due to compositional complexity and the sub-epidermal location of cells [34]. Moreover, it remains technically challenging to view the cell wall in a high throughput manner, and with enough resolution, to identify specific quantitative and qualitative changes in composition that directly accompany or precede changes in cellular identity. Dogma suggests that as cells divide into new microenvironments they are exposed to new combinations of hormones and signals, which subsequently activate receptors at the plasma membrane to cue signal cascades and downstream transcriptional changes [35,36]. As a result of this feedback, the cell wall is remodeled to introduce new or modified polymers that exhibit different properties and contribute to new cellular identity. This almost certainly involves changes in biomechanical properties, which have been extensively reviewed in recent times [37,38,39]. However, in order to receive and process a particular differentiation signal, what basic structural or biochemical features are required? Do specific polysaccharides or cell wall proteins enable the preferential accumulation of receptors, transmission of signals or the synthesis of signaling molecules that potentiate differentiation? Is there an ideal wall composition required for cell differentiation? Studies in recent years provide some answers, hinting that the cell wall plays a dynamic role in development, and that cues to initiate remodeling may arise from and depend on the composition of the wall itself. As mentioned above, recent reviews have considered in detail the role of cell wall integrity and sensors in controlling plant growth [40,41]. In this review, we consider molecular and genetic evidence supporting a role for distinct cell wall polysaccharides during plant development, particularly in light of recent studies and technological advances in cell-type specific transcriptional profiling. 

## 2. Cell Wall Modification during Growth, Differentiation, and Development

The molecular determinants of cell wall composition incorporate large families of enzymes including glycosyltransferases (GT), glycosylhydrolases (GH), methyltransferases, and acetylesterases (see the Carbohydrate Active enZyme database; CAZy [42]). The location and presumed site of activity of these enzymes can vary between the Golgi, the plasma membrane or a combination of both [43]. The addition of new polymers to a wall through the action of glycosyltransferases can immediately lead to changes in the pH, providing substrates for de-acetylation [44], de-esterification [19], and transglycosylation [45], and even new binding sites for receptors [46,47]. Specific differences in cell wall composition can be observed at different stages of development, between adjoining cells and tissues, and between monocots and dicots (See Figure 1). Several polymers that are labeled in Figure 1, pectin and callose, have been implicated in key stages of plant development. In the following sections we consider these polysaccharides, in addition to several “structural” polymers, with a view to addressing how their synthesis and/or modification can influence differentiation and development.

### 2.1. Pectin

**Figure 2:**
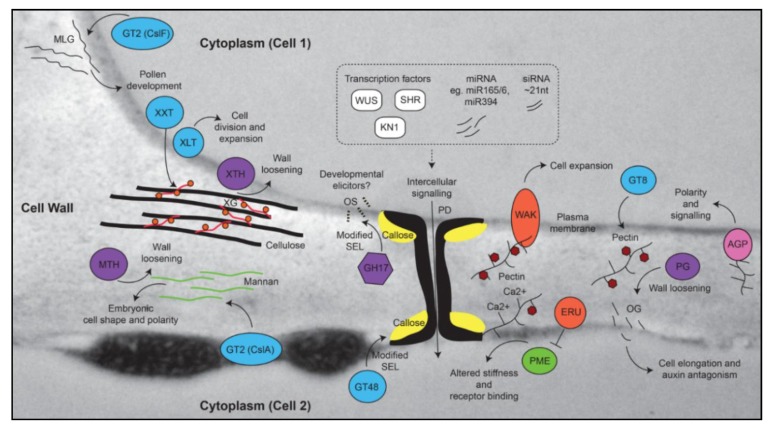
Cell wall components that contribute to growth, development, and differentiation. The model shows polymers superimposed on a TEM image of a leaf cell wall, including 1,3;1,4-β-glucan (MLG), cellulose, xyloglucan (XG), mannan, callose, and pectin. Enzymes that contribute to the biosynthesis or modification of these components are shown. The spatial separation of polymers is only shown for schematic purposes. Biosynthetic enzymes are shown in blue, hydrolytic enzymes are shown in purple, receptors are shown in orange, mobile transcription factors are shown in white, pectin methylesterase (PME) is shown in green, and arabinogalactan protein (AGPs) in pink. Deposition and hydrolysis of callose at the neck of plasmodesmata (PD) can alter the size exclusion limit (SEL) of the PD, hence limiting the mobility of intercellular signaling molecules such as transcription factors (e.g., WUSCHEL [59], SHORT ROOT [60], and KNOTTED [61]), microRNAs (miRNAs [60,62]), and short interfering RNAs (siRNAs [63,64]). Hydrolysis of callose by GH17 enzymes leads to the release of stimulatory oligosaccharides (OS) from the glucan backbone in fungi, but it remains unclear if similar OS contribute to growth and development in plants. By contrast, release of oligogalacturonides (OG) from pectin by polygalacturonase (PG) has been implicated in plant development through antagonistic effects on auxin pathways. The small circles on XG indicate galactosyl residues present due to the activity of XLT2 (xyloglucan galactosyltransferase). GT8 family enzymes contribute to the biosynthesis of pectin, which is usually synthesized in a methylesterified form (e.g., methylesterified homogalacturonan; meHG). Removal of methylesters (red hexagons) through the activity of PME can lead to calcium binding and subsequent cross-linking of pectin polysaccharides, which influences wall stiffness. GT, glycosyltransferase, XXT, xylosyltransferase, MTH, mannan transglycosylase/hydrolase, XTH, xyloglucan transglycosylase/hydrolase, CslF, cellulose synthase-like F, CslA, cellulose synthase-like A, GH, glycosyl hydrolase, WAK, wall-associated kinase, ERU, ERULUS receptor-like kinase.

Pectin is an important polymer during development since it can undergo considerable modification once it is deposited in the cell wall [48]. Multiple types of pectin are detected in the primary walls of dicots and monocots, including homogalacturonan (HG), rhamnogalacturonan-I (RG-I), rhamnogalacturonan-II (RG-II), and xylogalacturonan (XGA) [48,49]. Immunolabelling shows that pectic polymers are particularly enriched in young flowers, ovules, fruits, and roots (Figure 1). RG-I is detected in a number of tissues and is particularly prominent in the *Arabidopsis* seed coat [50] and the transition zone of developing roots [51]. The tight developmental regulation of RG-I deposition in seedling roots suggests it may play a role in cell expansion [51], but its exact role and the details of its biosynthesis remain unclear [52]. HG is methylesterified (meHG) during synthesis in the Golgi, and this forms a substrate for pectin methylesterase (PME, CE8), which depending on the cellular context can lead to loosening or strengthening of cell walls [19]. Clear roles for PME have been demonstrated in meristem development, seed mucilage biosynthesis, and pollen tube growth [53,54,55]. In the shoot meristem, organ primordia initiation requires demethylesterification of HG in sub-epidermal layers through the action of PME [56], which reduces stiffness and promotes outgrowth (Figure 2). Negative regulation of *PME5* in the meristem dome by the BELLRINGER transcription factor ensures that the meHG substrate is only targeted by PME5 at the flanks of the meristem, leading to correct positioning of organ primordial [37]. Similarly, in the root, alterations in PME activity and increased demethylesterification are associated with expansion of cell types in the root tip [57,58].

Other factors that influence cell expansion are the Wall-Associated Kinases (WAKs), which directly bind pectin polymers in the cell wall in a way that is at least partially dependent upon the degree of methylesterification [65,66] (Figure 2). Mutations in several WAK genes suggest they play a role in mediating resistance against various pathogens [67,68], as well as in cell expansion during development [69]. Another putative receptor involved in the pectin pathway is the *Arabidopsis Catharanthus roseus* receptor-like kinase 1-like (CrRLK1) ERULUS (ERU) protein, which is required for correct root hair formation, and regulates cell wall composition through negative control of PME activity [70] (Figure 2). Interestingly, ERU transcription is downregulated in several mutants showing changes in cell wall composition related to pectin, suggesting a possible feedback mechanism from the wall to regulate pectin composition and root hair development. ERU is part of the FERONIA (FER) family of kinases [41,71] that are implicated in fertilization, cell wall sensing, and root growth. Defects in the FER signaling pathway lead to pronounced defects in pectin composition of pollen tubes and root hairs, and a recent report indicates that FER directly interacts with pectin *in vivo* and in vitro [72]. Curiously, the ability of cell walls to sense change may be restricted to components of the primary wall, since limited signaling and transcriptomic responses were observed in mutants showing altered secondary cell wall biosynthesis in *Arabidopsis* [73].

Finally, modification of pectin by hydrolytic enzymes can lead to the release of small fragments called oligogalacturonides, which are reported to effect plant growth and development [74]. These pectin fragments impact diverse physiological processes, including fruit ripening in tomato [48] and stem elongation in pea [75] via a mechanism that appears to involve antagonism with the plant hormone auxin [76]. In summary, these studies indicate that specific pectic polymers within the wall may predispose cells to respond to stimuli that influence growth and differentiation.

### 2.2. Callose and Plasmodesmata

Another polymer that influences cellular differentiation is callose. Comprised of a water-insoluble linear form of (1,3)-β-glucan, callose is an atypical cell wall polysaccharide in that it is not often co-extensive throughout cell walls with pectin and cellulose but has specific restricted occurrences and functions in locations such as the cell plate, reproductive tissues, and plasmodesmata (PD). Genes involved in callose biosynthesis and hydrolysis are well characterized and include the 1,3-β-glucan synthases (GT48 family) and 1,3-β-glucan hydrolases (GH17 family), respectively. These enzymes have historically been associated with roles in pathogen response, dormancy, cell division, and plant reproduction [21,77,78], but recent studies emphasize their general importance in controlling intercellular transport of developmental regulators through PD (Figure 1 and Figure 2). PD are intercellular channels embedded in the cell wall that provide a cytoplasmic continuum between cells [79]. Different types of PD can be detected in the cell wall, which vary in terms of their structure and their arrangement within and between cell layers [80,81]. The formation of lateral roots in *Arabidopsis* depends upon restrictive callose deposits in the cell wall adjoining the PD [82], often referred to as the “neck” region. PD also regulate intercellular movement of transcription factors and microRNAs between the stele and endodermis to control xylem development [60]. Although the cues that drive PD formation are unknown, PD are present in many cell types and are accompanied by increased pectin and decreased cellulose deposits in flanking cell wall regions [83]. Enzymes regulating callose biosynthesis and turnover are enriched in the general PD proteome [84] in addition to several PMEs, polygalacturonases and diverse receptor kinases that likely influence PD function [85,86]. The biochemical analysis of PD highlights a potential relationship between pectin and callose that has yet to be explored in significant detail.

The removal of callose from PD and specialized cell walls in the anthers and ovule is mediated by GH17 enzymes, which form a large family found in archaea, bacteria, and eukaryotes [87]. In general, GH17 activity is likely to influence growth and development in several ways by (1) decreasing the size exclusion limit (SEL) of PD and allowing increased symplastic intercellular transport [88]; (2) removing apoplastic barriers that are proposed to insulate cells such as the megaspores or microspores against mobile signals [89,90] and (3) removing a transient matrix for deposition of secondary polymers during cytokinesis and cell division [91]. Consistent with a role in regulating the SEL of PD, studies in the shoot meristem have shown that mobile tracers are free to move between distinct “symplastic fields”, which incorporate different zones and layers [92,93]. This indicates that differential regulation of PD conductance is likely to be required for meristem cell identity and function. One key transcription factor involved in meristem maintenance, WUSCHEL, moves from the organizing centre (OC) of the meristem into above-lying stem cells through PD [59]. Therefore, the presence of PD and associated cell wall polymers is another example by which cells may be predisposed to be responsive to non-cell autonomous stimuli; in essence, the PD and adjoining regions of cell wall provide a substrate for receptor binding as well as for cell wall remodeling activities that can influence intercellular signaling and differentiation (Figure 2).

In addition to these developmental functions, GH17 enzymes also form a defensive barrier during pathogen attack that targets 1,3-β-glucan polymers in the fungal cell wall. A recent study showed that non-branched fungal 1,3-β-glucan oligosaccharides are able to trigger immune responses in *Arabidopsis* via CERK1 (chitin elicitor receptor kinase 1) [94]. It is tempting to speculate that similar to oligogalacturonides, cleavage of endogenous 1,3-β-glucan polymers might release backbone oligosaccharides that elicit responses during growth and development (Figure 2). 

### 2.3. Roles for Other “Structural” Polymers in Growth and Development

1,3;1,4-β-glucan is predominantly found in monocots, particularly the *Poaceae*, where it accumulates in the primary and secondary walls of diverse tissues [95,96] (Figure 1). Evidence suggests that accumulation of 1,3;1,4-β-glucan is required for correct grain fill in barley and wheat [97,98]. However, genetic studies also reveal specific developmental abnormalities, such as male infertility, in rice plants lacking the primary biosynthetic enzyme controlling 1,3;1,4-β-glucan biosynthesis [99] (Cellulose synthase-like F6; CslF6). In barley, tissue-specific over-accumulation of 1,3;1,4-β-glucan appears to inhibit signal and/or solute transmission [97,29] while barley *cslf6* mutants are shorter and show defects in leaf growth [100]. This is perhaps unsurprising given that *CslF6* is expressed in a range of tissues [101], however, the specific role of 1,3;1,4-β-glucan and the *CslF* gene family in plant development requires further investigation. 

Unlike 1,3;1,4-β-glucan, xyloglucan (XyG) is a highly branched polysaccharide found in the primary cell wall of many plant tissues and is characterized as a structural cell wall component that binds to cellulose [102] (Figure 2). Remarkably, mutants lacking activity of three xylosyltransferase (*XXT*) genes (*XXT1*, *2* and *5*) contain no detectable xyloglucan in their cell walls, yet develop relatively normally apart from defects in root hairs [103]. By contrast, *murus3* mutants that are deficient for a XyG-specific galactosyltransferase contain normal levels of xyloglucan, but in a form that is depleted of galactosyl substituents, and this results in extreme developmental defects including dwarfism [104]. Hence, while XyG is not required per se for *Arabidopsis* development, incorrect substitution of XyG may compromise interactions between different wall polymers, resulting in a cell wall composition that is incompatible with cell growth.

Similar to xyloglucan, several types of structurally diverse mannans are also linked to the cellulose network providing mechanical support [105], while others are involved in carbohydrate storage. Loss-of-function mutations in the *Cellulose synthase-like A* (*CslA*) 2, 3, and 9 genes, encoding putative glucomannan synthases, result in no detectable glucomannan in stems but plants appear phenotypically normal [106]. However, mutants lacking function of the *CslA7* gene show embryo lethality, suggesting that in some tissues glucomannan is a critical component for growth and differentiation [107]. Although the mechanistic basis for this lethality is unclear, the *csla7* mutant embryos appear remarkably similar to those showing defects in developmental patterning and organ differentiation, such as double mutants of the *WUSCHEL-HOMEOBOX* 8/9 transcription factors [108] and *ARGONAUTE* 1/10 genes involved in post-transcriptional gene silencing [109,110]. This may indicate that targets of these transcriptional and post-transcriptional regulators converge at the cell wall, or that a distinct cell wall composition contributes to downstream function of these regulatory pathways.

Interestingly, both mannan and xyloglucan are targets of transglycosylase enzymes activities, which essentially cleave the polysaccharide chain and attach it to a new chain to retain strength in the cell wall (Figure 2). Both mannan endotransglycosylases/hydrolases (MTH) and xyloglucan endotransglycosylases/hydrolases (XET/XTH) have been implicated in fruit development. LeMAN4a, an MTH from tomato, exhibits transglycosylase activity and is expressed in young floral buds where it is hypothesized to function in tissue softening [111]. Similarly, *XTH* genes are associated with fruit development in persimmon, apple, and tomato [112,113]. Therefore, even in the case of polysaccharides that have historically been associated with structural functions, there is evidence to suggest their presence in the wall may provide a substrate for remodeling enzymes that impact growth and differentiation during diverse stages of plant development.

## 3. Specific Cell Wall-Related Genes Accompany Differentiation in Meristematic Zones

Antibodies and glyco-arrays are an outstanding resource [114,115] to localize and identify specific cell wall-related epitopes, and this is highlighted by the distinct labelling patterns shown in Figure 1. The limitation of antibodies is that they only provide a limited view of the chemical complexity present in a cell wall at a particular time point. Technologies that enable local qualitative and quantitative assessments of wall complexity, particularly in the case of the shoot and root meristem and reproductive tissues, would provide a significant advantage in understanding cell wall changes during differentiation. Methods such as coherent anti-Stokes Raman scattering (CARS [1]) and FTIR microspectroscopy [116] may enable specific compositional changes to be identified, although they are yet to deliver the required precision for cell-type specific analysis during development. By contrast, at the molecular level, definition of the transcriptional programs underlying cell wall formation has recently become much more accessible. The analysis and identification of cell wall-related genes that define specific cell types and/or show altered expression during development remains a viable approach to assess the role of different cell wall components in facilitating differentiation.

In *Arabidopsis*, studies have utilized the elegant method of fluorescence-assisted cell sorting (FACS) to collect specific populations of cells from developing tissues [117,118,119]. This approach was used successfully in *Arabidopsis* roots [117,118] to profile RNA from, among others, cell types located in the meristematic zone including the quiescent centre (QC), the adjoining columella, and the lateral root cap (LRC). The QC is marked by the expression of *AGL42* and *WOX5* genes and contains slowly dividing, “undifferentiated” cells that stimulate the formation of adjoining stem cells [120] (Figure 1). Underlying the QC are the columella initials; stem cells that divide periclinally to give rise to one daughter that adopts columella fate and another that retains stem cell identity. Similarly, the LRC initial cells adjoin the QC and give rise to all cells in the lateral root cap. These cell types are in close proximity but assume different identities as soon as they divide away from the QC. Therefore, the cell-type specific transcriptional datasets provide an excellent resource to assess changes in the cell wall machinery during differentiation.

**Figure 3:**
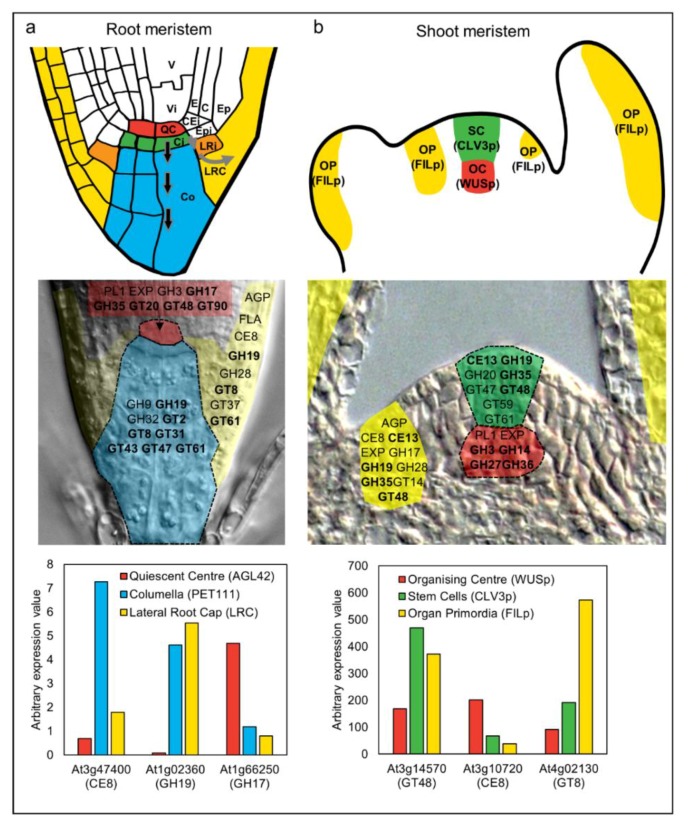
Analysis of cell wall-related gene expression during differentiation of stem cells in the root and shoot meristem of *Arabidopsis thaliana*. The upper panels in (**a**,**b**) show schematic representations of the root and shoot apical meristem [120]. (**a**) In the root meristem, initial cells (stem cells) directly adjoining the QC enter differentiation pathways as they divide away from the niche (shown by arrows for columella and lateral root cap). V, vasculature, Vi, vascular initial, QC, quiescent centre, E, endodermis, C, cortex, CEi, cortex/endodermis initials, Epi, epidermal initials, Ep, epidermis, LRi, lateral root cap initial, LRC, lateral root cap, Ci, columella initial, Co, columella. (**b**) In the shoot meristem, the organizing center (OC) functions via WUSCHEL (WUS) to maintain the stem cells (SC) in an undifferentiated state. The stem cells express the signal peptide CLAVATA3 (CLV3). Divisions of the stem cells provide daughters that enter differentiation pathways at the flanks of the meristem and become organ primordia (OP), which is marked by expression of genes such as *FILAMENTOUS FLOWER* (*FIL*). The second row of panels highlights gene families encoding CAZy carbohydrate-related enzymes [42] that are enriched in each meristem cell type according to FACS-mediated sorting and transcriptional profiling [118,119]. The genes are superimposed on sections of root and shoot meristem tissues. Family names in bold indicate that multiple members from the same family were up-regulated in the QC or OC (depending on the meristem) relative to both of the other cell types. GH, glycosyl hydrolase, GT, glycosyltransferase, PL, pectate lyase, AGP, arabinogalactan protein, EXP, expansin, CE, carbohydrate esterase, FLA, fasciclin-like arabinogalactan protein. See Table 1 for putative functions of enzyme families. The third row of panels shows expression patterns of selected CAZy family members in the different meristem cell types. Several of the individual genes reflect the behavior of the entire family. For example, At1g02360 is up-regulated in the columella and LRC relative to the OC, and this is a pattern shown for many GH19 family members. However, other genes such as At3g47400, At3g10720, and At4g02130 show unique patterns compared to other members of their families. The reason why multiple family members are recruited into some cell-type preferential expression pathways, while in others only individual members are expressed, remains to be elucidated.

Houston et al. [4] examined transcriptional datasets from *Arabidopsis* and other species to highlight cell wall gene families associated with cell wall remodeling during abiotic stress and pathogen attack. A similar survey of the *Arabidopsis* root cell-type specific RNA profiles [118] reveals a comprehensive set of cell wall genes potentially contributing to growth and differentiation (Figure 3). Relative to the QC (as an undifferentiated reference), cells that adopt LRC or columella fate express different gene families involved in polysaccharide biosynthesis and modification. Examples include the arabinogalactan proteins (AGPs), pectin methylesterases (CE8), glucoronyl/galacturonosyltransferases (GT8), and xylan 1,4-β-xylosyltransferases (GT43). Arabinogalactan proteins are cell wall proteins that have been implicated in many aspects of growth and development [30,31,121,122], while the other families are implicated in pectin and xylan biosynthesis and modification. The majority of these gene families are upregulated as cells adopt columella or LRC identity, consistent with the formation of new wall types compared to the relatively naïve wall in the undifferentiated QC. Notably, within the QC itself, representatives from the pectate lyase (PL1), expansin, 1,3-β-glucanase (GH17), and 1,3-β-glucan synthase (GT48) families are up-regulated, hinting at a key requirement for intercellular signaling and wall flexibility. This analysis exemplifies how transcriptomic studies can enable identification of cell wall-related genes and families that accompany changes in cell identity during differentiation. In many cases, these transcriptional changes directly relate to alterations in root cell wall composition [123] indicating a close link between transcript abundance and putative enzyme activity.

In the shoot apical meristem (SAM), Yang et al. (2016) characterized changes in cell wall composition by immunolabelling, in addition to profiling cell wall-related gene expression in different meristematic regions [124]. Their results indicate that as cells divide through the meristem, different enzymes build new walls compared to those that build maturing walls. Complementing this, studies have examined transcriptional changes at the level of individual meristematic cell types (Figure 3). The organizing centre (OC) of the SAM is marked by expression of the *WUSCHEL* gene and is somewhat similar to the root QC, in that it is undifferentiated, slow to divide, and specifies adjoining cells as stem cells [120]. The shoot stem cells express the *CLAVATA3* gene, and as they divide anticlinally, they exit the control of the OC and enter organ differentiation pathways where expression of transcription factors such as *FILAMENTOUS FLOWER* are detected (Figure 3). Yadav et al. (2009) used these cell-type specific markers to isolate and transcriptionally profile shoot stem cell types [119]. Around half of the *Arabidopsis* CAZy cell wall families are up-regulated in organ primordia but downregulated in the stem cells relative to the OC; gene families include the expansins (EXP), fasciclin-like arabinogalactan proteins (FLAs), pectate lyases (PL1), pectin methylesterases (CE8), polygalacturonases (GH28), and endo-arabinanases (GH43). The lack of glycosyltransferases and abundance of cell wall modifying enzymes suggests that, similar to the root meristem, cell wall remodeling is the predominant feature of cell and organ differentiation in the shoot. Interestingly, gene families that are up-regulated in the stem cells relative to the OC and organ primordia include a number of key polysaccharide synthases and hydrolases such as 1,3-β-glucan synthase (GT48), arabinosyl/xylosyltransferase (GT61), and xylanase (GH10). As discussed above, the GT48 genes contribute to callose biosynthesis, and their up-regulation may relate to the formation of symplastic zones through altered PD conductance. Although a direct role for GT61 and GH10 genes during development has not been explicitly reported, GT61 enzymes have been implicated in substitution of polysaccharides to potentially influence wall polymer viscosity in seed-coat epidermal cells [125,126], and some GH10 xylanases are expressed during secondary wall synthesis in poplar [127]. 

In summary, these studies show that as cells exit the stem cell niche and start differentiating, clear trends are seen in the transcriptional behavior of CAZy families. The CAZy signatures of distinct cell-types within the shoot and root meristem are summarized in Figure 3. It is important to note that despite their grouping via functional domains and proposed carbohydrate-related activities, the vast majority of the CAZy genes remain uncharacterized. The transcriptional profiles of the meristematic cells are remarkably dynamic yet similar between the shoot and root meristems, identifying key activities whose role in differentiation might be addressed in more detail through further mutant and cell-type specific analyses.

## 4. Perspectives

The basis for this review was to consider the role of the plant cell wall in growth and development, and to assess how cell wall polysaccharides might predispose cells to undergo differentiation. We have focused our attention on polysaccharides including pectin, callose, xyloglucan, and mannan, which fulfil roles during different stages of growth and development. The presence and modification of these polymers correlates with changes in cell identity and function, and their depletion through mutagenesis or transgenic modification results in altered plant development. Callose and pectin in particular provide multiple avenues to influence differentiation, initially through deposition and subsequently through hydrolysis, chemical modification, and receptor binding. Consistent with the chemical complexity of the cell wall, the transcriptional machinery underlying cell wall polysaccharide deposition and modification is intricate. However, common activities are identified in cell types that exit from apical (shoot and root) stem cell niches and initiate differentiation. This overlap suggests that while the cellular context (i.e., roots vs. shoots) and specific gene family members might differ, early stages of differentiation likely depend on a similar wall composition that is compatible with remodeling. In this context, it seems prudent to consider the cell wall in the same light as other key factors, such as genomic and epigenetic modifications, that facilitate important steps of the cell differentiation process.

## Figures and Tables

**Table 1:** Protein families potentially involved in polysaccharide biosynthesis and modification in *Arabidopsis*.

CAZy Family	Putative Polysaccharide Target	Gene ID	Enzyme Description
AGP			arabinogalactan protein *
CE13	Pectin		pectin acetylesterase
CE8	Pectin	PME	pectin methylesterase
EXP			expansin
FLA			fasciclin-like arabinogalactan protein
GH3	Glucan/Xylan/Xyloglucan		β-d-glucosidase, α-l-arabinofuranosidase, β-d-xylopyranosidase
GH5	Mannan	MTH	endo-β-mannanase
GH9	Cellulose		cellulase
GH10	Xylan		endo-β-xylanase
GH14	Starch		β-amylase
GH16	Xyloglucan	XTH/XET	xyloglucan:xyloglucosyltransferases
GH17	Callose	GLUC	glucan endo-1,3-β-glucosidase
GH19	Chitin		chitinase; lysozyme
GH20			beta-hexosaminidase
GH27			α-galactosidase
GH28	Pectin	PG	polygalacturonase
GH32			invertase
GH35	Pectin/Xyloglucan		β-galactosidase
GH36			α-galactosidase
GT2	Cellulose/Mannan/1,3;1,4-β-glucan	CslA/CslF	cellulose synthase/cellulose synthase-like
GT8	Pectin/Xylan		homogalacturonan 1,4-α-galacturonosyltransferase UDP-GlcA: xylan α-glucuronyltransferase
GT14	AGP		UDP-GlcA: [arabinogalactan] 1,3-β-/1,6-β-galactan 1,6-β-glucuronosyltransferase
GT20			alpha,alpha-trehalose-phosphate synthase [UDP-forming]
GT31	AGP/Pectin		1,3-β-glucuronyltransferase
GT34	Xyloglucan	XXT	xyloglucan 1,6-α-xylosyltransferases
GT37	Xyloglucan		xyloglucan 1,2-α-α-fucosyltransferase
GT43	Xylan		glucuronoxylan glycosyltransferase
GT47	Xylan/Xyloglucan	MUR3	xylosyltransferase/xyloglucan galactosyltransferase
GT48	Callose	GSL	1,3-β-glucan synthase
GT59			1,2-α-glucosyltransferase
GT61	Xylan/Xyloglucan		xylosyltransferase/arabinosyltransferase
GT90	Mannan		UDP-Xyl: (mannosyl) glucuronoxylomannan galactoxylomannan 1,2-β-xylosyltransferase
PL1	Pectin		pectate lyase

Note: * AGPs are not reported to exhibit enzymatic activity. Only families relevant to Figure 2 or the main text are included while genes that are referred to in the text are listed in the Gene ID column.
